# A comparison of three types of targeted, community-based methods aimed at promoting early detection of new leprosy cases in rural parts of three endemic states in India

**DOI:** 10.1371/journal.pone.0261219

**Published:** 2021-12-14

**Authors:** Karthikeyan Govindasamy, Annamma S. John, Vivek Lal, Mohammad Arif, Raju Moturu Solomon, Jyoti Ghosal, Ambarish Dutta

**Affiliations:** 1 Research Domain, The Leprosy Mission Trust India, New Delhi, India; 2 German Leprosy and Tuberculosis Relief Association (GLRA India), Kolkata, West Bengal, India; 3 Netherlands Leprosy Relief India, New Delhi, India; 4 Regional Medical Research Centre, Indian Council of Medical Research, Bhubaneswar, Orissa, India; 5 Indian Institute of Public Health, Public Health Foundation of India, Bhubaneswar, Orissa, India; The University of Georgia, UNITED STATES

## Abstract

**Background:**

India achieved elimination of leprosy nationally in 2005, but since then the number of patients with grade 2 disability at diagnosis increased steadily indicating delay in diagnosis. Therefore, there was a need for public health interventions which can increase case finding in their earlier stage. The objective of this study is to compare the effectiveness of three such community-based interventions; 1) Enhancement of community awareness on leprosy; 2) Education and motivation of “Index” leprosy cases; and 3) Involvement of Non-Formal Health Practitioners (NFHPs) to promote early detection of new cases of leprosy.

**Methodology/principal findings:**

Three community-based interventions were implemented between April 2016 and March 2018, embedded within the National Leprosy Eradication Program (NLEP) of India. Interventions were 1) increasing awareness through involvement of Gram Panchayat (local government) in the community regarding early signs of leprosy (Awareness), 2) providing health education and motivating newly diagnosed leprosy patients to bring suspects from their contacts (Index) and 3) training local non-formal health practitioners (NFHP). Each intervention was implemented in a group of ten blocks (sub-division of district) with an additional ten blocks as control (with no intervention). The main outcomes were number of new cases detected and number of grade 2 disability among them. They were obtained from the routine NLEP information system and compared between these interventions. On an average, there was an addition of 1.98 new cases in Awareness blocks, 1.13 in NFHP blocks and 1.16 cases in Index intervention blocks per month per block after adjusting for changes in control blocks during the same period. In terms of ratio, there was a 61%, 40% and 41% increase in case notification in awareness, Index and NFHP intervention, respectively. Overall, the percentage of grade 2 disability across intervention blocks declined.

**Conclusion:**

The Awareness intervention appears to be more effective in detection of new cases, compared to Index case motivation and sensitization of NFHPs. However, it is important to stress that while selecting strategies to increase early diagnosis it is important to determine, which is the most appropriate for each context or area and must be decided depending on the local context.

## Introduction

Globally, in the year 2019, India contributed 57% of all new cases of leprosy detected [1]. Despite the declaration of ‘elimination of leprosy as a public health problem’ (Prevalence Rate of less than 1 per 10,000 population) from the country in December 2005, a number of high endemic districts persist in few Indian states. During the decade (April 2008- March 2009 to April 2018-March 2019) a substantial number of new cases were detected each year ranging from 120,334 to 135,485 without any sign of significant decline [2]. Although there was a decline in the proportion of child cases among new cases during the same decade from 10.1% to 7.7%, the actual numbers remained high, indicative of continuing transmission of leprosy [3]. As on March 2014, when this research project was developed 198/657 (30%) districts in the country notified an annual new case detection rate (ANCDR) of more than 10 per 100,000 populations. In addition, the proportion of new cases with grade 2 disability rose steadily from 1.87% in 2005 to 4.61% in 2015, suggesting a considerable delay in diagnosis and reporting of new cases, detected through the routine surveillance system. Against this backdrop and given the fact that most of the districts registering a high case burden were predominantly rural and economically backward, experts opined various probable reasons for the continuing transmission and delay in detection [4, 5], that included 1) leprosy patients seeking initial care from the private informal health system rather than the public health system in which leprosy diagnosis and treatment capacity often remained insufficient, 2) inadequate contact tracing of other members of the household and immediate neighbourhood (often related to leprosy-associated stigma), 3) delay in care seeking due to dwindling knowledge and awareness of leprosy symptoms and its consequences in the community [6, 7], as the previous health education division of the erstwhile leprosy programme had been dismantled following the declaration of elimination in 2005. Therefore, there was a strong felt-need for public health interventions to be urgently conceptualized de novo, redesigned and/or repurposed from other similar disease-control programmes that have the potential to increase case finding and ensure earlier detection of leprosy cases. This was necessary to address the shortcomings mentioned above that leads to continuation of transmission and detection delays; and at the same time be sustainable, scalable and not resource intensive.

### The project

Consequently, an implementation research project was conceptualized by The Leprosy Mission Trust India (TLMTI) along with two other partner Non-Government Organizations, Netherlands Leprosy Relief-India and German Leprosy & Tuberculosis Relief Association–India (GLRA India), and in close consultation with National Leprosy Eradication Programme (NLEP), to try out three different community-based interventions within the existing NLEP framework, aimed at promoting early detection of new leprosy cases. These interventions were 1) Enhancement of community awareness on leprosy; 2) Education and motivation of “Index” leprosy cases to identify suspects among their contacts and refer to appropriate health facility; and 3) Involvement of Non-Formal Health Practitioners (NFHPs) to identify and refer patients with signs and symptoms of leprosy. The following sub-sections describe these three arms of intervention.

#### Enhancement of community awareness of leprosy

This arm (hereinafter referred to as “Awareness” arm) comprised ‘Community Awareness on leprosy sessions, which were organised by the project staff with the cooperation and involvement of the local NLEP staff. The standardized sensitization sessions were all conducted in the local language and lasted for about 90 minutes. The sessions included a talk on signs and symptoms of leprosy, importance of treatment and regularity and recognition of complications of leprosy. The awareness programs were conducted for each Panchayat (the local self-government at the lowest that is the village-level) in the administration system of rural India, usually in the Panchayat building or in the nearby public facilities such as public schools, offices or library.

#### Education and motivation of the Index leprosy case

In this arm (hereinafter referred to as the “Index” arm), an index case was defined as a patient who has been newly diagnosed with leprosy in the study area, at a health facility operated either by the public health system or partner NGOs such as The Leprosy Mission Trust India. The health facility staff sensitized the index cases during the routine health education given to new cases regarding the early signs of leprosy, significance of regular and complete treatment and prevention of disability. In addition, the importance of screening of contacts of leprosy affected persons [8, 9] and how to conduct such screening was the focus of the interaction with the index cases in this arm. Subsequently, the index cases were encouraged to examine all the contacts from both family and non-family for early signs of leprosy and refer the suspects to health facility for diagnosis.

#### Involvement of Non-formal Health Providers (NFHP)

This arm is hereinafter referred to as “NFHP” arm. In India NFHPs are often the first person, especially in less-developed rural areas, to whom anyone with a health problem contacts for advice [10, 11]. They mostly practice western allopathic medicine, albeit with no formal training, therefore, mostly inappropriately. In addition, some of them also practice traditional medicine and faith healing. They cater to a large section of the rural population, especially early in the stages of their ailments of any kind. The project staff identified and enrolled the well-attended NFHPs in the blocks (sub-division of district in rural areas with population of 100,000 or more). All the enrolled NFHPs were then sensitised through structured orientation sessions focussing on importance of regular treatment and prevention of disability, early and suggestive signs and symptoms of leprosy and when and where to refer the suspects they come across in their practices. Standardized training modules were used across all sites. All the training sessions were conducted in the local language. Training of the NFHPs, was facilitated by the Investigator and Co-investigators with domain expertise in clinical aspects of leprosy. The staff from the block level public health system and NLEP were also involved in trainings as co-facilitators to promote sustainability after the end of the project.

**Fig 1:**
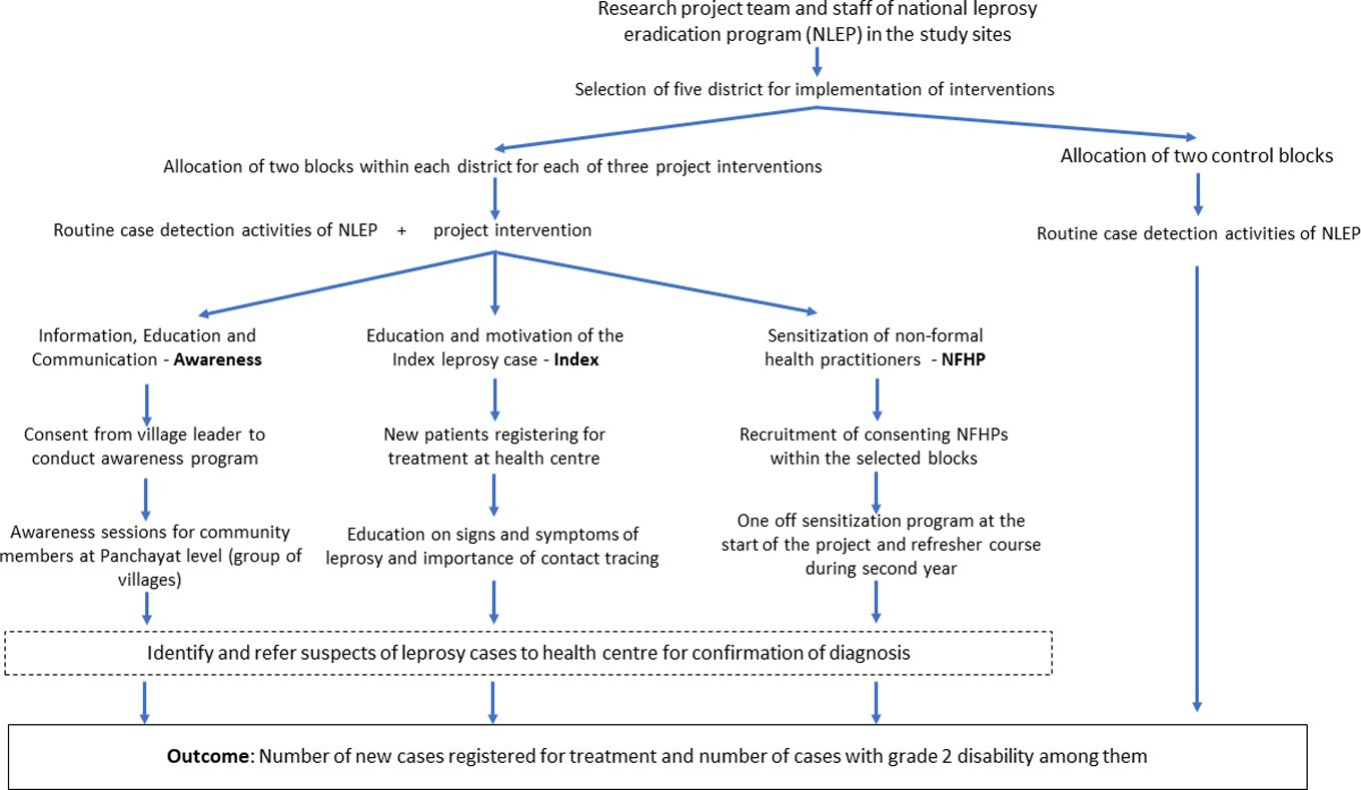
The flow diagram of study process.

All new leprosy cases diagnosed through these three arms were notified to and reported through the routine information system of NLEP. All those new cases suspected through awareness and NFHP interventions were provided with referral slips which were used to determine the intervention upon confirmation of diagnosis. Those suspects identified through contacts of Index cases were either brought by the index case for examination or they informed project/NLEP staff over phone. Upon registration, the intervention name through which they were identified, was recorded on the patient card. Further description on interventions is given in S1 Appendix. The overall study process is shown as flow diagram in Fig 1.

### Selection of study sites

**Fig 2:**
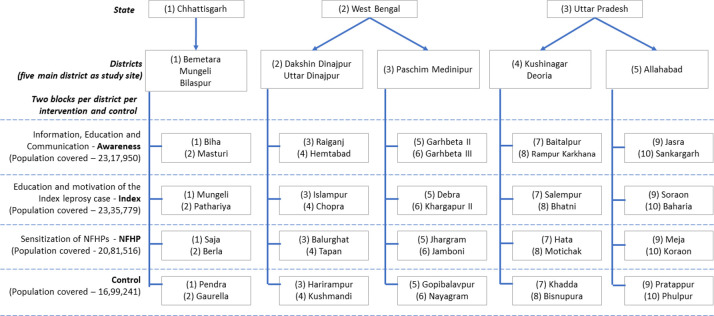
Study sites with population covered.

Three states, which were known to record high number of cases prior to this research project, were selected as study area. The selected states were West Bengal (WB), Chhattisgarh (CG) and Uttar Pradesh (UP). From the selected states, five main districts were selected as study sites, two in WB, two in UP and one in CG. The unit of intervention was blocks within the district. A block is a sub-division of district, the Indian administrative structure in rural areas with approximately 100,000 or more population. Two blocks from each of the five districts received one of the three interventions, therefore, six blocks from each district were considered as intervention blocks. Additionally, two blocks from each district were selected as “control” where no intervention was applied, but the routine NLEP activities were carried out. When sufficient number of blocks were not available in the selected main district, blocks from adjacent district were selected as study area. The Fig 2 illustrates the selected three states, five main districts and two blocks for each intervention and two blocks as control area. Therefore, each intervention was implemented in group of ten blocks and additional ten blocks as control. The districts were selected in consultation with NLEP of the country and the states; and as per the operational convenience of the other stakeholders of the study. An effort was made to ensure that the boundaries of the blocks, where a specific intervention was planned, were not contiguous with blocks where a different intervention was planned.

### Objective

To assess and compare the impact of the three different interventions; 1) Enhancement of community awareness on leprosy; 2) Education and motivation of “Index” leprosy cases; and 3) Involvement of Non-Formal Health Practitioners (NFHPs) to promote early case detection using a quasi-experimental research analysis framework.

## Materials and methods

### Ethics statement

This study was approved by The Leprosy Mission Trust India Ethics Committee before the commencement of the study. The reference number of the approval letter is EC070415 No:6/15/a. For each intervention, aggregated data was obtained from the NLEP monthly reports, therefore we did not take consent from individual patients, except for patients in Index arm where oral consent was taken from individual patients before giving them education about screening and referral of their contacts. At the community level, before the commencement of the study, local leaders such as village head and other Panchayat Raj leaders (local government) were consulted, and the purpose of the study explained to obtain their permission. Oral consent was taken from all the non-formal health practitioners before including them in the sensitization program of the study. No individual patient identity was obtained as part of data collection or analysis.

### Data collection

#### Process data of the project

During the intervention period total number, of suspects of leprosy cases identified and number of confirmed new leprosy cases among them from each intervention arm, were collected to study the yield in the number of new cases detected. The confirmation of new cases was based on WHO definition of diagnosis, by medical officer in the health centre as per standard protocol of National Leprosy Eradication Program. After examination of suspects of leprosy and he/she considered new case when presence of at least one of the following cardinal signs: 1) definite loss of sensation in a pale or reddish skin patch; 2) a thickened or enlarged peripheral nerve with loss of sensation and/or weakness or muscles supplied by that nerve; and/or 3) presence of acid-fast bacilli in a slit-skin smear. The source of the data was the information system of the project where these data were collected every month per block and consolidated according to the intervention. The main inclusion criteria were that the new suspects and confirmed new cases of leprosy should be from the respective selected area for intervention or control area. The data on those coming from outside the intervention area were excluded from the data collection.

#### Effectiveness of the interventions

The primary outcome of the study comprised block-wise total number of new cases registered in a month along with the number of new cases with different grades of disability according to WHO disability grading system of 0, 1 and 2 [12]. The source, of the data, was routine NLEP block-level monthly reports and two hospitals of The Leprosy Mission Trust India in the two study sites in UP and CG, which were collected by the project staff every month from every study block, intervention as well as control. The data were set up as block-wise monthly time-series from April 2013 up to March 2018.

As per the analysis plan adopted in this study, only block-level aggregated data were used, and no individual-level data were collected or used for any purpose.

### Sample and power calculation

The sample size was calculated as per the study design for a quasi-experimental study with block as the unit of intervention. The level of significance testing (Type I error) was set at 0.05, the power (Type II error) of the study was set at 80%, the incidence of leprosy in the population was reported to be 10/100,000/year (from 2014–15 national report of NLEP) and the expected change after intervention was set at 40%. The design effect to account for clustering of blocks within districts and to control for intra-community correlation and community-level variation was set to 4. The calculated sample size for each arm of intervention came to approximately 800,000, which justified selection of ten blocks for each intervention strategy. The average population of a block is approximately 100,000; hence over 1 million people were in each of the community-based intervention arms.

### Statistical analysis

#### Process data of the project

The descriptive statistics was used to study the yield and additionality from each intervention. The percentage of confirmed new leprosy cases against suspects of leprosy, identified from each intervention, is reported as yield. The number and percentage of new leprosy cases reported through our intervention among total cases registered in NLEP during the project period is reported as additional new cases added because of the effect of the project interventions. The one-way ANOVA test was used to test the difference between the intervention for average confirmation rate (confirmed new cases against those identified with signs that are suggestive of leprosy) and average additional cases added at block level. Two-sided *p*-value of <0.05 was considered statistically significant.

#### Effectiveness of the interventions

The primary outcome measures included total number of new leprosy cases and number of new leprosy cases with grade 2 disability among them at the time of diagnosis–a proxy measure of time elapsed between disease onset and its diagnosis. Difference-in-difference (DID) analysis using a Poisson regression model was done to test the effectiveness of each intervention by comparing the change in monthly average cases notified during pre and intervention period after adjusting for changes in the control blocks during the same period. The pre-intervention period being April 2013 to March 2016 and intervention period being April 2016 to March 2018. The changes were reported as ratio of cases between these two periods, also called Risk Ratio (RR). The DID estimates were reported as RRs of each intervention arm minus that in the control arm. We also calculated the percentage of patients with grade 2 disability among new cases to study its changes over time. Unexpectedly, the NLEP led active leprosy case detection campaign (LCDC) was carried out during the intervention period in three out of five sites of the study area. The campaign aimed to actively search new cases through door-to-door survey, the methodology of the campaign is available elsewhere [13–15]. The LCDC took place in three out of five project sites, two in West Bengal and one in Chhattisgarh in two rounds per year for two years: first round between April to June and second round between September to November. During the first year (2016–17) of our intervention there were two rounds in both the states. During the second year (2017–18), there were two rounds in Chhattisgarh and one round in West Bengal. The details of the timing and the total cases detected during LCDC campaign is shown in S1 Table. Therefore, to study the effect of our intervention the outcome was analysed as pooled (all five sites) and stratified, based on the sites where LCDC was carried out (three sites) and the sites where no such active case detection campaign was done (two sites). We calculated 95% confidence interval for Difference-in-difference in monthly average of new cases notified and risk ratio (RR). The one-way ANOVA test was used to test the difference in average number of new cases detected each year (pre-intervention and intervention period) and number of grade 2 disability among them. Two-sided *p*-value of less than 0.05 was considered statistically significant. The statistical analysis was performed using R statistical software 4.1.0 (2021-05-18)—"Camp Pontanezen" [16].

## Results

### Process outcome of the project

Table 1 shows the extent of interventions, number of suspects of leprosy identified and confirmed cases against suspects. During the intervention period 1,610 community awareness programmes were conducted, 23,031 community members attended the awareness program. On an average 15 to 20 people attended each IEC session in a village based on our count, which excludes those people standing and watching the IEC activity whose number is not practically possible to count. After awareness intervention 377 (31%) new cases of leprosy were confirmed out of 1,233 suspects identified through this intervention. A total 1021 index cases were educated and of them 809 suspects among their contacts were referred to health centres by them for diagnosis. Of all those examined 256 (32%) were confirmed as new leprosy cases. A total of 1247 NFHPs were sensitized on leprosy, and they referred 672 suspects of leprosy, of which 137 (20%) were confirmed to be leprosy in the health centre. The difference in average confirmation rate at block level between the three intervention arms was found to be not statistically significant (p-value, 0.368).

**Table 1:** Summary of number of cases suspected and confirmed among suspects from the three intervention areas.

Group	Intervention	Suspects identified	Confirmed cases against suspects (%)	P-value
Awareness	1610 awareness programs conducted	1233	377 (31)	0.368
Index	1021 Index cases educated	809	256 (32)	
NFHP	1247 NFHPs sensitized	672	137 (20)	

The summary of effectiveness of intervention blocks and control blocks is shown in Table 2. Of the 1,466, 1,198 and 1,153 cases reported in NLEP from three different intervention blocks during the intervention period, 377, 256 and 137 of them were detected through our awareness, Index and NFHP interventions, respectively. The percentage of additional cases added through our intervention arms were high for awareness arm (25.7%), followed by Index arm (21.5%) and NFHP arm (11.9%). The difference in average additional cases added at block level between the three intervention arms was statistically significant (p-value, <0.001). All three interventions added significantly higher number of additional cases over and above the number detected through routine program activity.

**Table 2:** Summary of number of new cases identified through our interventions among new cases registered in NLEP during the intervention period.

Interventions	Approximate population covered	Total NLEP registered Cases from Intervention Blocks	Number (%) of new case identified through our intervention among total registered in the NLEP	P-value
Awareness	23,17,950	1466	377 (25.7)	<0.001
Index case	23,35,779	1198	256 (21.4)	
NFHPs	20,81,516	1153	137 (11.9)	
Control area	16,99,241	669	No intervention	-

### Effectiveness of intervention

Table 3 shows the change in average monthly new case notifications per block and its rate ratio across three arms during the intervention period as compared to pre intervention period, adjusted for the changes in the control area. In the pooled analysis, the change of 0.95 case increase was not statistically significant. When stratified according to LCDC and non-LCDC area, there was an addition of two cases (1.98) in the non-LCDC area in the awareness intervention blocks, statistically significant (p-value, <0.05). Although there is an increase in Index (0.11) and NFHP (0.13) intervention blocks in the pooled analysis, the yield was not statistically significant. When stratified, there was an increase in the average number of cases in both the arms, 1.13 in NFHP and 1.16 in Index, in the non-LCDC area but the yield was not statistically significant. In terms of ratio, there was a 61%, 40% and 41% increase in case notification in awareness, Index and NFHP arms, respectively, in the non-LCDC areas (p-value, <0.05). The similar increase was not observed in the pooled and LCDC areas.

**Table 3:** Difference-in-difference in monthly average of new cases notified during intervention period as compared to pre intervention period across intervention blocks after adjusting for control blocks and its ratio.

Strata	Phase	Difference-in-difference in monthly average of new cases notified (95% CI)			Risk Ratio (95% CI)		
		Awareness	Index	NFHP	Awareness	Index	NFHP
Pooled data	Pre	Ref	Ref	Ref	Ref	Ref	Ref
	Post	0.95 (-0.20,2.11)	0.11 (-0.92,1.13)	0.13 (-0.85,1.11)	1.02 (0.90, 1.16)	0.89 (0.78, 1.01)	0.90 (0.79, 1.03)
LCDC	Pre	Ref	Ref	Ref	Ref	Ref	Ref
	Post	0.27 (-1.20, 1.74)	-0.57 (-1.87, 0.71)	-0.55 (-1.78, 0.68)	0.81 (0.69, 0.94)	0.71 (0.61, 0.82)	0.72 (0.62, 0.83)
No LCDC	Pre	Ref	Ref	Ref	Ref	Ref	Ref
	Post	1.98 (0.30, 3.65)*	1.13 (-0.31, 2.57)	1.16 (-0.20, 2.51)	1.61 (1.32, 1.96)*	1.40 (1.15, 1.71)*	1.41 (1.16, 1.73)*

* Statistically significant.

The change in average monthly new case with grade 2 disability at diagnosis among all new cases notified per block and its rate ratio across three arms during the intervention period as compared to the pre intervention period, adjusted for the changes in the control area is shown in Table 4. In the pooled analysis, on an average, the reduction in the G2D cases was higher in awareness arm (-0.030), followed by NFHP (0.008) and least in the Index (0.003) intervention blocks. When stratified, the trend in the reduction among intervention blocks were similar in both LCDC and non-LCDC blocks. However, the reduction observed was not statistically significant.

**Table 4:** Difference-in-difference in monthly average percentage of grade 2 disability among new cases notified during intervention period as compared to pre intervention period across intervention blocks after adjusting for control blocks and its ratio.

Strata	Phase	Difference-indifference in monthly average percentage of G2D among new cases notified (95% CI)			Risk Ratio (95% CI)		
		Awareness	Index	NFHP	Awareness	Index	NFHP
Pooled data	Pre	Ref	Ref	Ref	Ref	Ref	Ref
	Post	-0.030 (-0.17, 0.11)	0.008 (-0.10, 0.11)	-0.008 (-0.09, 0.08)	1.21 (0.59, 2.58)	1.378 (0.65, 2.99)	1.13 (0.50, 2.58)
LCDC	Pre	Ref	Ref	Ref	Ref	Ref	Ref
	Post	-0.027 (-0.20, 0.14)	0.011 (-0.12, 0.14)	-0.005 (-0.10, 0.09)	1.39 (0.54, 4.08)	1.59 (0.60, 4.71)	1.30 (0.47, 4.0)
No LCDC	Pre	Ref	Ref	Ref	Ref	Ref	Ref
	Post	-0.035 (-0.25, 0.18)	0.003 (-0.16, 0.16)	-0.013 (-0.13, 0.10)	1.49 (0.41, 2.9)	1.19 (0.45, 3.36)	0.98 (0.35, 2.8)

The increase in number of new cases detected and reduction in the percentage of patients with grade 2 disability at the time of diagnosis among all new cases notified in a year during pre-intervention and intervention period is shown in Table 5. In the pooled analysis, the number of new cases increased during the intervention period as compared to pre-intervention period. The average increase in the number of new cases at block level between the years was statistically significant for all three intervention arms and in control area. Overall, the percentage of grade 2 disability decreased across intervention blocks as well as in the control blocks during the intervention period as compared to pre-intervention years. However, the average decrease in the grade 2 disability at block level between the years was not statistically significant. The pattern was similar in the LCDC and non-LCDC areas, more so in the latter area.

**Table 5:** Total number of new leprosy cases detected and percentage of G2D disability among them during intervention and pre intervention period, stratified by the LCDC campaign.

Intervention	LCDC campaign	Pre-intervention						Intervention				P-value	
		2014		2015		2016		2017		2018			
		New	G2D	New	G2D	New	G2D	New	G2D	New	G2D	New	G2D
**Awareness**	Pooled	520	35 (6.7)	558	29 (5.2)	523	37 (7.1)	789	30 (3.8)	677	8 (1.2)	0.0243	0.0535
	LCDC	439	32 (7.3)	485	29 (6.0)	433	34 (7.9)	676	25 (5.9)	501	7 (1.8)	-	-
	No LCDC	81	3 (3.7)	73	0	90	3 (3.3)	113	5 (4.4)	176	1 (0.6)	-	-
**Index**	Pooled	452	16 (3.5)	568	31 (5.5)	483	25 (5.2)	710	19 (2.7)	488	12 (2.4)	0.0158	0.118
	LCDC	354	14 (4.0)	467	35 (7.5)	374	18 (4.8)	634	15 (3.3)	411	12 (3.7)	-	-
	No LCDC	98	2 (2.0)	101	6 (5.9)	109	7 (6.5)	76	4 (5.2)	77	0	-	-
**NFHP**	Pooled	473	18 (3.8)	536	17 (3.2)	418	16 (3.8)	669	17 (2.5)	484	8 (1.7)	0.0203	0.474
	LCDC	351	13 (3.7)	415	15 (3.6)	319	12 (3.8)	606	10 (2.3)	424	6 (1.8)	-	-
	No LCDC	122	5 (4.1)	121	2 (1.7)	99	4 (4.0)	63	7 (11.1)	60	2 (3.3)	-	-
**Control**	Pooled	260	14 (5.4)	259	8 (3.1)	229	8 (3.5)	372	8 (2.2)	297	3 (1.1)	0.0346	0.44
	LCDC	137	8 (5.8)	161	5 (3.1)	136	3 (2.2)	282	4 (2.4)	208	0	-	-
	No LCDC	123	6 (4.9)	98	3 (3.1)	93	5 (5.4)	90	4 (4.4)	89	3 (3.3)	-	-

## Discussion

In India, after the declaration of elimination of leprosy as a public health problem in 2005, the voluntary reporting has been a major strategy in detection of new leprosy patients in the absence of regular active detection campaigns. Various methods such as population survey, school survey and training of multipurpose health workers were the main modes of active case detection before 2005. Yet, there is a lack of evidence on effective methods to promote early detection of leprosy which is sustainable and not so resource intensive. The objective of the project was to find effective case detection methods to encourage early reporting in the community. To evaluate the effectiveness of early case detection, we analysed the number of new leprosy cases notified and the number of people with G2D disability among them. The decreasing number of G2D is an indication of the awareness about leprosy in the community, capacity of health staff to diagnose leprosy and quality of leprosy services to some extent [17]. The rising number of new cases notified with lower number of G2D indicates the early detection of the cases [18–20].

In the pooled analysis (Table 3) of Awareness arm, although the absolute number of cases notified was increased the difference was not statistically significant, perhaps due to inclusion of cases detected through LCDC in both intervention and control blocks, therefore, the expected difference-in-difference diminished so in the sites where LCDC was held. The effect of our intervention was apparent in the sites where no LCDC was carried out. On an average there was an addition of 0.98 cases (95% CIs 0.30 to 3.65) which was 61% (95% CIs, 32% to 96%) increase in number of cases per block notified in a month after our intervention. During the corresponding period, there was a decrease in the absolute number of cases with G2D disability among new cases, but it was not significant (Table 4). Nevertheless, there was an apparent decreasing trend in the percentage of G2D, from 7.1% in baseline to 1.3% during the second year of intervention (Table 5), indicating early detection and diagnosis. These findings clearly support the effectiveness of Awareness intervention. Raising awareness about leprosy in the community found to be increasing the number of new cases detected in their early stage in other endemic parts of India [21, 22] and globally [17]. The pattern of initial increase in number of new cases observed is consistent with the expected trend when community awareness level raises or an active case detection was carried out [18, 23]. All the cases were confirmed and registered for treatment by the health workers of the NLEP which validates the diagnosis in every new leprosy case.

In the second intervention, we motivated all the newly diagnosed patients and educated them about early signs of leprosy. Through this intervention there was an increase of 41% (15% to 71%) in a month per block in non-LCDC area, supporting the effectiveness of the intervention. The effect was not observed in pooled analysis and in the LCDC sites (Table 3). There was a decreasing trend in the proportion of G2D from 5.2% at baseline to 2.4% during the second year, indicating the early detection of new cases (Table 5). A total of 809 contacts of Index cases were referred with suggestive signs of leprosy to the health centre, of whom 256 were confirmed to be suffering from leprosy, which is 21.4% of total new cases (1,198) reported during the study period in the Index arm area (Table 2). The percentage, of contacts diagnosed (32%) among index cases (Table 1), was slightly higher than other studies from other developing countries, (23%) in Nepal [24] and (25%) in Bangladesh [25]. Also, the number of suspects identified by Index patients is less than one per index cases, given the fact that contacts of index cases are at higher risk of contracting the infection [8, 26–29]. This is perhaps because of the methodology adopted in the study. We restricted ourselves to the education and motivation of index cases at health centre only so that they could examine their own contacts and refer the suspects. Home visits could have given an opportunity for better examination and covering all the contacts, therefore, identification of more cases, but would put a strain on the workers and might not be carried out regularly. Home visits also could trigger resistance from the index patients to examine their contacts due to disclosure of diagnosis and possible resulting stigma [7, 30–32].

In the third intervention, we sensitized 1247 non-formal health practitioners (NFHPs) during the study period. They were sensitized on signs and symptoms of leprosy to refer those suspected to have leprosy for diagnosis. Through them 672 suspected cases were referred to heath centre of whom 137 were confirmed to be suffering from leprosy which is 11.9% of total cases (1,153) detected in the intervention area (Table 2). Increase in case detection was on an average 41% (95% CIs, 16% to 73%) in a month per block during intervention in the non-LCDC area. The similar gain in detection of new cases was not apparent in pooled analysis and in LCDC areas (Table 3). During the corresponding period, in the pooled analysis the proportion of G2D among new cases fell from 3.8% to 1.7% at the end of intervention, suggesting the early detection of cases (Table 5). The referral by NFHP was much less than expected considering reports from previous studies that they are the first point of contact for health needs in general [10, 33] and for the leprosy related health needs of the local population [34, 35]. We conducted informal interviews with the NFHPs to understand the reason for the low referrals by them. The main reason was found to be the hesitation to refer to government health facility as they are not the recognized practitioners by the government. Providing them with the incentive based on each confirmed case among those referred could increase the referrals from them.

The limitation of the study was that the effectiveness of intervention was confounded by the LCDC activities implemented by the government in the study sites including control areas [13, 14]. The LCDC was announced and carried out after the project was already in progress and there was no way to compensate for its effects. The LCDC activities of both phases in 2017 and 2018 had a direct effect on the project outcome. They are as follows: 1) This drive resulted in the detection of large number of new cases which were detected all over India, and particularly in high endemic states, including our study sites and has reduced the number of new cases we had been expecting from our interventions, because those cases were already detected by the campaign. This is apparent from our pooled analysis and data from the sites where LCDC campaign was implemented. Therefore, results in the study sites where no LCDC was implemented can be considered as an underpowered sub-sample of the study as the expected results were diluted by the LCDC. In spite of this, there is still an impact, though not always statistically significant for all interventions. 2) Another effect has been that with this sudden rise in cases in some study sites, the field investigators were unable to motivate all new cases immediately on diagnosis, as per our protocol (in the Index case motivation). An indirect effect of the campaign was that during the campaign period, all the health department work forces were geared to implement the campaign activities which has reduced the voluntarily reported cases in the NLEP during the campaign months. This has influenced the expected clear increasing trend in new cases and corresponding decrease in the proportion of G2D in the intervention area. It is important to stress that the LCDC campaign is a massive effort and very resource intensive and cannot be sustained. This supports the interventions tested in this study which can be taken forward by the NLEP utilising general health staff depending on the epidemiological status of leprosy in their area. The LCDC also introduced the possible bias that majority of the undetected cases in the community were detected through campaign even in the control, therefore, we do not find much difference between intervention and control area. As a result, effort interventions were neutralized by the LCDC.

We recommend sustained IEC campaign in the national leprosy program which can lead to increase yield. Index and NFHP didn’t work as much as we expected. Perhaps Index case couldn’t convince suspects to reach hospital or suspects was not identified by NFHP or suspects didn’t reach NFHP. The reason for low detection in Index and NFHP to be studied and address in the future studies. The future studies can test implementing all three interventions in the same area. Given the long incubation period of the disease and studies of this nature would take at least 5 to 6 tears to show significant impact on new case detection and interruption of transmission. Hence, the future studies should aim to follow-up for longer duration.

In conclusion, the Awareness intervention appears to be more effective in detection of new cases, compared to Index case motivation and NFHP intervention. However, it is important to stress that while selecting strategies to increase early diagnosis it is important to determine which is the most appropriate for each context or area and must be decided depending on the local context.

## Supporting information

S1 Appendix Description of interventions. (DOCX)

S1 Table Number of cases detected through LCDC campaign and their timings. (DOCX)
