# Supplementary material for: A comparison of three types of targeted, community-based methods aimed at promoting early detection of new leprosy cases in rural parts of three endemic states in India
Source: PLoS One. 2021 Dec 14;16(12):e0261219. doi: 10.1371/journal.pone.0261219 (PMC8670664; doi:10.1371/journal.pone.0261219)
Supplement: S1 Appendix — (DOCX) [file pone.0261219.s001.docx]

**S1 Appendix - Description of interventions**

**A comparison of three types of targeted, community-based methods aimed at promoting early detection of new leprosy cases in rural parts of three endemic states in India**

**Name of the intervention**:

1. Awareness - Increasing community awareness through involvement of Gram Panchayat (local government at village level) in the community regarding early signs of leprosy.
2. Index – Providing health education and motivating newly diagnosed leprosy patients to bring their contacts suspected lesions of leprosy.
3. NFHP – Training local non-formal health practitioners (NFHP) on identification of signs and symptoms of leprosy and referral to health facility for diagnosis and treatment.

**Methodology of implementation of intervention:**

The standard operating procedures were developed for individual interventions in consultation with all the partners of the project and respective district level national leprosy program staff. The individual interventions were implemented according to the agreed SOPs.

**Awareness:** The baseline awareness level about leprosy was established in the intervention blocks through survey and found to be low. We used banner with the photos of early signs and symptoms of leprosy with the caption in the local language. This banner was put up in each awareness program which was held in each Gram Panchayat in a common place where everyone can meet together.

The banners used in the awareness program was developed in consultation with patient’s community, staff of national leprosy program and stakeholders of this project. Initially, appropriate permissions were obtained from the Gram Panchayat (local government) leaders before conducting awareness program in a particular area and sought their involvement. The awareness programs were conducted along with the national leprosy program staff in most instances. The program was designed to be delivered in 60 to 90 mins with the structured sequence of points covering early signs and symptoms of leprosy and its complications and emphasize was given that the leprosy is curable with multi-drug therapy and encouraged the community to report or refer anyone who is suspected to be suffering from leprosy to nearby health centre for diagnosis and treatment.

It took for about 12 to 15 months to cover all the Panchayats. The program was scheduled in such a way that there will be 2 to 3 program per week which varied depending on the availability of facilitators. The interventions were delivered in the local language of the study sites. In Hindi language in two sites of the Uttar Pradesh, Chhattisghari and Hindi in the one site in Chhattisgarh and in Bengali in two sites in West Bengal.

***Index***: The new patients who were registered for treatment were visited during their second or third visit. The short education session was provided by national leprosy program staff or project staff covering following points; leprosy is curable, caused by germs, close contacts may develop leprosy, early signs of leprosy as patches, weakness of hands, feet and eyes and motivated them to examine their contacts and bring or refer to local health facility of those with signs of having leprosy for diagnosis. The Index case education was done for all those newly diagnosed and registered for treatment in the respective intervention blocks. The intervention continued throughout the intervention period at least giving one month to examine their contacts of those recruited during the final two months of the intervention period.

***NFHP***: The project staff enumerated all the NFHPs in the intervention blocks. All the NFHPs were contacted and informed them about the aim of the project and encouraged them to participate. All those consented were called to common place for sensitization program. The facilitators for the sensitization programs were from district leprosy officers, medical officers of the health centre from the intervention area and staff of national leprosy program and experts from the TLMTI, NLR and GLRA. The program lasted for 120 to 180 minutes covering basic knowledge about signs and symptoms of leprosy to enable them to suspect early cases among those coming to them with such symptoms and refer to the appropriate health centre. The project staff were regularly in contact with the NFHPs through phone call to encourage them to continue to be part of this project. One year later follow-up sensitization program was conducted to enhance their knowledge. The NFHP sensitization program were conducted during the first or second months of the first year of the intervention period and follow-up program at the beginning of second year of the intervention.

**Provider of the intervention**: All three interventions were provided by staff employed in this project along with the staff of the national leprosy program. During the later period of the intervention all the interventions were conducted by the staff of national leprosy program.

**Mode of delivery of the intervention**: All the interventions were provided face to face manner. The awareness program was conducted in a group with variable numbers depending on the response from the local area where the program was conducted. The index case education was provided in one to one session. The NFHP sensitization program was provided in a group. Each study site conducted their sensitization program separately for their respective NFHPs.

**Fidelity of the intervention**: The SOPs were prepared prior to implementation of the intervention. The awareness program had structured format which was followed across all five sites. The common curriculum for the sensitization program for NFHPs were followed across all study sites. The points to be covered in the index case education was prepared as part of SOPs as checklist and the person who is educating the index patients expected to cover all the points. To monitor the progress of the interventions monthly reporting format was developed and reviewed every month.

**Monitoring of the intervention:** Program manager made regular field visits to ensure that the SOPs are followed as planned. The monthly reports were discussed every month by the program manager and the principal (ASJ) and co-investigator (KG) and feedback was given to project staff with instructions to change anything if required. Six monthly and yearly review were done along with representative from partners site to ensure that the SOPs are followed as planned and if required new amendments were made in the SOPs to improve the intervention outcome. For example, in the sites where the active case detection campaigns were held, we decided to halt the awareness program during that particular month as all the national leprosy program staff were involved in the campaign.
