# Supplementary material for: A comparison of three types of targeted, community-based methods aimed at promoting early detection of new leprosy cases in rural parts of three endemic states in India
Source: PLoS One. 2021 Dec 14;16(12):e0261219. doi: 10.1371/journal.pone.0261219 (PMC8670664; doi:10.1371/journal.pone.0261219)
Supplement: S1 Table — (DOCX) [file pone.0261219.s002.docx]

S1 supplementary table: **Number of cases detected through LCDC campaign and their timings.**

| **State** | **Timing** | **LCDC campaign rounds in a year** | **Number of cases detected during the campaign** | **Total cases detected during the reporting year** |
| --- | --- | --- | --- | --- |
| Chhattisgarh | May 2016 | 1 | 44 | 287 |
|  | Sep to Nov 2016 | 2 | 243 |  |
|  | Jun 2017 | 1 | 19 | 109 |
|  | Sep to Oct 2017 | 2 | 90 |  |
| West Bengal | Apr to Jun 2016 | 1 | 46 | 439 |
|  | Sep 2016 | 2 | 393 |  |
|  | Nov 2017 | 1 | 274 | 274 |

The LCDC took place in three out of five project sites, two in West Bengal and one in Chhattisgarh in two rounds: first round between April to June and second round between September to November. During the first year (2016-17) of our intervention there were two rounds in both the states. During the second year (2017-18), there were two rounds in Chhattisgarh and one round in West Bengal.
